# Favorable survival outcomes in epidermal growth factor receptor (EGFR)-mutant non-small cell lung cancer sequentially treated with a tyrosine kinase inhibitor and osimertinib in a real-world setting

**DOI:** 10.1007/s00432-023-04839-3

**Published:** 2023-05-18

**Authors:** Oliver Kraskowski, Jan A. Stratmann, Marcel Wiesweg, Wilfried Eberhardt, Martin Metzenmacher, Kurt W. Schmid, Thomas Herold, Hans-Ulrich Schildhaus, Kaid Darwiche, Clemens Aigner, Martin Stuschke, Katharina Laue, Gregor Zaun, Stefan Kasper, Jörg Hense, Martin Sebastian, Martin Schuler, Michael Pogorzelski

**Affiliations:** 1 Department of Medical Oncology, West German Cancer Center, University Hospital Essen, Hufelandstr. 55, 46147 Essen, Germany; 2 Department of Internal Medicine, Hematology/Oncology, University Hospital Frankfurt, Frankfurt, Germany; 3 Institute of Pathology, West German Cancer Center, University Hospital Essen, Essen, Germany; 4 German Cancer Consortium (DKTK), Partner Site University Hospital Essen, Essen, Germany; 5 Section of Interventional Pneumology, Department of Pneumology, West German Cancer Center, University Medicine Essen-Ruhrlandklinik, Essen, Germany; 6 Department of Thoracic Surgery and Surgical Endoscopy, West German Cancer Center, University Medicine Essen-Ruhrlandklinik, Essen, Germany; 7 Department of Radiation Oncology, West German Cancer Center, University Hospital Essen, University Duisburg-Essen, Essen, Germany; 8 German Cancer Consortium (DKTK), Partner Site University Hospital Frankfurt, Frankfurt, Germany

**Keywords:** Sequential therapy, Osimertinib, Rebiopsy, EGFR-mutated NSCLC, EGFR T790M mutation, Second line

## Abstract

**Purpose:**

EGFR tyrosine kinase inhibitor (TKI) therapy in EGFR-mutated lung cancer is limited by acquired resistance. In half of the patients treated with first/second-generation (1st/2nd gen) TKI, resistance is associated with EGFR p.T790M mutation. Sequential treatment with osimertinib is highly active in such patients. Currently, there is no approved targeted second-line option for patients receiving first-line osimertinib, which thus may not be the best choice for all patients. The present study aimed to evaluate the feasibility and efficacy of a sequential TKI treatment with 1st/2nd gen TKI, followed by osimertinib in a real-world setting.

**Methods:**

Patients with EGFR-mutated lung cancer treated at two major comprehensive cancer centers were retrospectively analyzed by the Kaplan–Meier method and log rank test.

**Results:**

A cohort of 150 patients, of which 133 received first-line treatment with a first/second gen EGFR TKI, and 17 received first-line osimertinib, was included. Median age was 63.9 years, 55% had ECOG performance score of ≥ 1. First-line osimertinib was associated with prolonged progression-free survival (*P* = 0.038). Since the approval of osimertinib (February 2016), 91 patients were under treatment with a 1st/2nd gen TKI. Median overall survival (OS) of this cohort was 39.3 months. At data cutoff, 87% had progressed. Of those, 92% underwent new biomarker analyses, revealing EGFR p.T790M in 51%. Overall, 91% of progressing patients received second-line therapy, which was osimertinib in 46%. Median OS with sequenced osimertinib was 50 months. Median OS of patients with p.T790M-negative progression was 23.4 months.

**Conclusion:**

Real-world survival outcomes of patients with EGFR-mutated lung cancer may be superior with a sequenced TKI strategy. Predictors of p.T790M-associated resistance are needed to personalize first-line treatment decisions.

**Supplementary Information:**

The online version contains supplementary material available at 10.1007/s00432-023-04839-3.

## Introduction

Approximately, 12–15% of non-squamous non-small cell lung cancers (NSCLC) are molecularly characterized by an activating mutation in the epidermal growth factor receptor (EGFR) (Cancer Genome Atlas Research 2014; Rosell et al. 2009; Zhang et al. 2016). Tyrosine kinase inhibitors (TKI) are the optimal first-line treatment of patients with stage IV NSCLC harboring TKI-sensitive EGFR mutations (Maemondo et al. 2010; Ramalingam et al. 2020; Rosell et al. 2012; Sequist et al. 2013; Wu et al. 2014, 2017). FDA- and EMA-approved first generation (1st gen) TKI erlotinib and gefitinib bind solely and reversibly to EGFR, whereas second generation (2nd gen) TKI afatinib and dacomitinib bind irreversibly to EGFR, HER2 and HER4, which highlights relevant molecular and clinical differences between 1st and 2nd gen TKIs (Li et al. 2008; Robichaux et al. 2021). The clinical benefit of TKI treatment is generally limited by the inevitable development of acquired resistance, which is associated with the EGFR p.T790M gatekeeper mutation in approximately 50–60% of patients treated with 1st or 2nd gen EGFR TKI (Yu et al. 2013). 3rd gen TKI osimertinib was specially designed to target EGFR p.T790M resistance mutation (Cross et al. 2014). In patients who developed p.T790M resistance mutation, a sequential treatment with osimertinib is highly active (Mok et al. 2017; Papadimitrakopoulou et al. 2020). Recently, first-line osimertinib was approved based on superior progression-free survival (PFS) in comparison to 1st gen EGFR TKI (Soria et al. 2018). A survival benefit for first-line osimertinib was also reported, but 30% of patients received no second-line treatment and data are still immature (Ramalingam et al. 2020). There is no approved sequential targeted therapy for patients progressing on first-line osimertinib and chemotherapy/immunotherapy is of limited activity (Hastings et al. 2019; Lee et al. 2017; Lisberg et al. 2018; Mok et al. 2017; Papadimitrakopoulou et al. 2020). Hence, first-line treatment with a 1st/2nd gen EGFR TKI followed by osimertinib at confirmation of p.T790M-mediated resistance may be a superior strategy in some patients. Here, we explored the outcome of a sequential treatment approach in a real-world patient population treated at two German comprehensive cancer centers.

## Materials and methods

### Study design and patients

Patients with EGFR-mutated NSCLC treated with 1st, 2nd or 3rd gen TKIs at the West German Cancer Center (WTZ), University Hospital Essen and the University Tumor Center (UTC), University Hospital Frankfurt, between January 2008 and January 2021 were included. All treatment and follow-up data were documented in the electronic health records (EHR). Clinico-pathological parameters, treatment trajectories and outcomes were also retrieved from the EHR. The data cutoff for follow-up was March 17, 2021. The study was approved by the Ethics Committees of the Medical Faculty of the University Duisburg-Essen and the University of Frankfurt (19-8585-BO).

### Clinical assessments

Routine staging procedures included a whole-body (18)F-fluorodeoxyglucose positron-emission tomography (FDG-PET-CT) or computed tomography (CT) scans of the chest and abdomen, and brain magnetic resonance imaging (MRI). Tumor staging was based on the 8th Edition of the UICC/WHO staging system. Under TKI treatment, radiological assessments were performed every 10–12 weeks according to the institutional guidelines of the WTZ and UTC. The response rate was retrospectively evaluated according to the Response Evaluation Criteria in Solid Tumors 1.1 (RECIST 1.1) (Eisenhauer et al. 2009; Therasse et al. 2000). Response assessment was feasible if at least baseline and one follow-up imaging dataset were available. Overall survival was defined as time from first administration of palliative TKI treatment to death from any cause. Patients were censored at the time of last follow-up, if time of death was unknown. Progression-free survival (PFS) was defined as time from the start of TKI therapy to date of radiologic progression or death.

### Statistical analysis

Statistical analyses were performed using SPSS Statistics (V26, IBM, Armonk, NY, USA) and MS Excel 2010 (VS 14.0, Microsoft, Richmond, WA, USA). Survival analyses were performed by the Kaplan–Meier method and log rank test. Comparison of p.T790M acquisition was analyzed through Chi-square test for categorical data.

## Results

### Patients’ characteristics

A total of 150 patients with EGFR-mutated NSCLC treated in first line with 1st, 2nd or 3rd gen TKI were included in this analysis. Baseline clinical characteristics are summarized in Table 1. The median age was 63.9 years (range 35.7–87.4) with 32.0% (*N* = 48) of patients older than 70 years at the start of the first TKI. Notably, 82 patients (54.7%) had an ECOG performance status of 1 or higher. More patients were female (65.3%, *N* = 98), never/light smokers with a smoking history of less than 10 pack years (63.3%, *N* = 95). *EGFR* exon 19 in-frame deletions were the most prevalent mutations (58.7%, *N* = 88). A total of 112 patients (74.7%) were initially diagnosed with systemic disease (stages IV A/B), whereas 38 patients initially had stage I–III disease (25.3%).

**Table 1:** Baseline clinical characteristics and therapy

			%	*N*
Median age			63.9 (range 35.7–87.4)	
Age > 65 years			50.0	75
Age > 70 years			32.0	48
Age > 75 years			18.0	27
Age > 80 years			6.7	10
Gender, female			65.3	98
ECOG				
ECOG 0			40.7	61
ECOG 1			46.0	69
ECOG 2			7.3	11
ECOG 3			1.3	2
Not documented			4.7	7
Stage at primary diagnosis (IASCL/UICC 8th Edition)				
	I		4	6
	II		2.6	4
	III		16.7	25
	IVA	M1a	16.7	25
		M1b	8.7	13
	IVB		51.3	77
Stage at the start of palliative TKI				
	IVA	M1a	26.7	40
		M1b	12.0	18
	IVB		61.3	92
Primary histology				
Adenocarcinoma			98.7	148
Adenosquamous carcinoma			1.3	2
EGFR mutation	Del exon 19		58.7	88
	Exon 21(L858R)		29.3	44
	Uncommon		12.0	18
Smoking status	Never		43.3	65
	< = 10 py		20.0	30
	11–29		10.0	15
	> = 30		14.0	21
	n.d		12.7	19
Curatively intended treatment			25.3	38
Surgery			81.6	31
Chemoradiotherapy			18.4	7
Palliative radiotherapy			63.3	95
First TKI				
1st generation TKI			39.3	59
	Erlotinib		22	33
	Gefitinib		17.3	26
2nd generation TKI				
	Afatinib		49.3	74
3rd generation TKI				
	Osimertinib		11.3	17

### First-line treatments

All patients initially diagnosed with stage I–III disease were initially treated with curative intent, which is also detailed in Table 1. 1st gen TKI (erlotinib or gefitinib) was administered as first-line TKI in 59 patients (39.3%), 74 patients (49.3%) received first-line treatment with afatinib, and 17 patients (11.3%) received osimertinib as their first-line TKI.

### Outcomes of first-line treatments

**Fig. 1:**
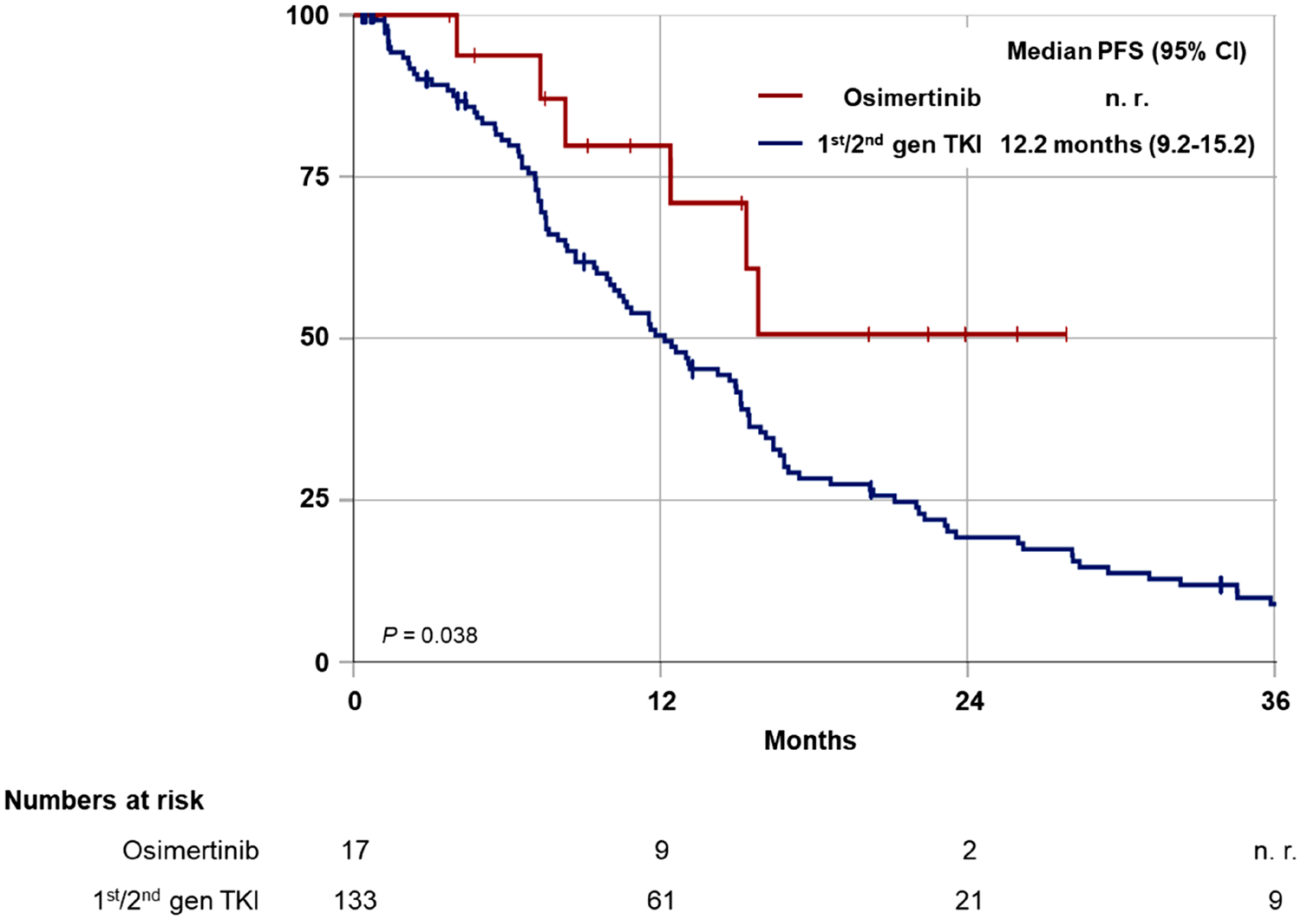
Median progression-free survival (PFS) in patients with EGFRmt NSCLC from start of first TKI for first-line 1st/2nd gen TKI vs osimertinib

**Fig. 2:**
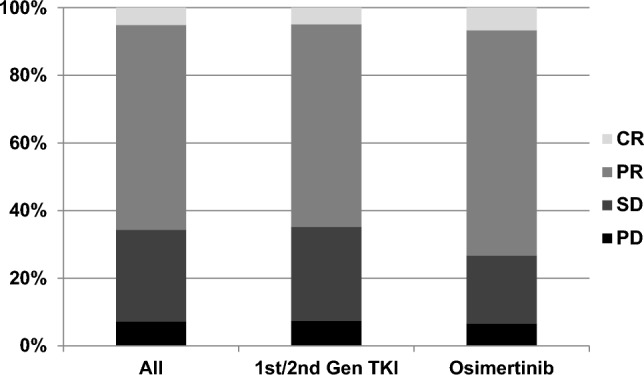
Response rate (RR) and disease control rate  (DCR) for 1st/2nd gen TKI vs osimertinib

Progression-free survival under first-line TKI treatment was superior in patients receiving osimertinib as compared to 1st/2nd gen TKIs (median not reached vs 12.2 months, *P* = 0.038) (Fig. 1). In total, 137 patients (91.3%) were evaluable for response according to RECIST 1.1. Response rates (RR) and disease control rates (DCR) for first-line osimertinib and 1st/2nd gen TKIs were 73.3% vs. 64.8% and 93.3% vs. 92.6%, respectively (Fig. 2).

**Fig. 3:**
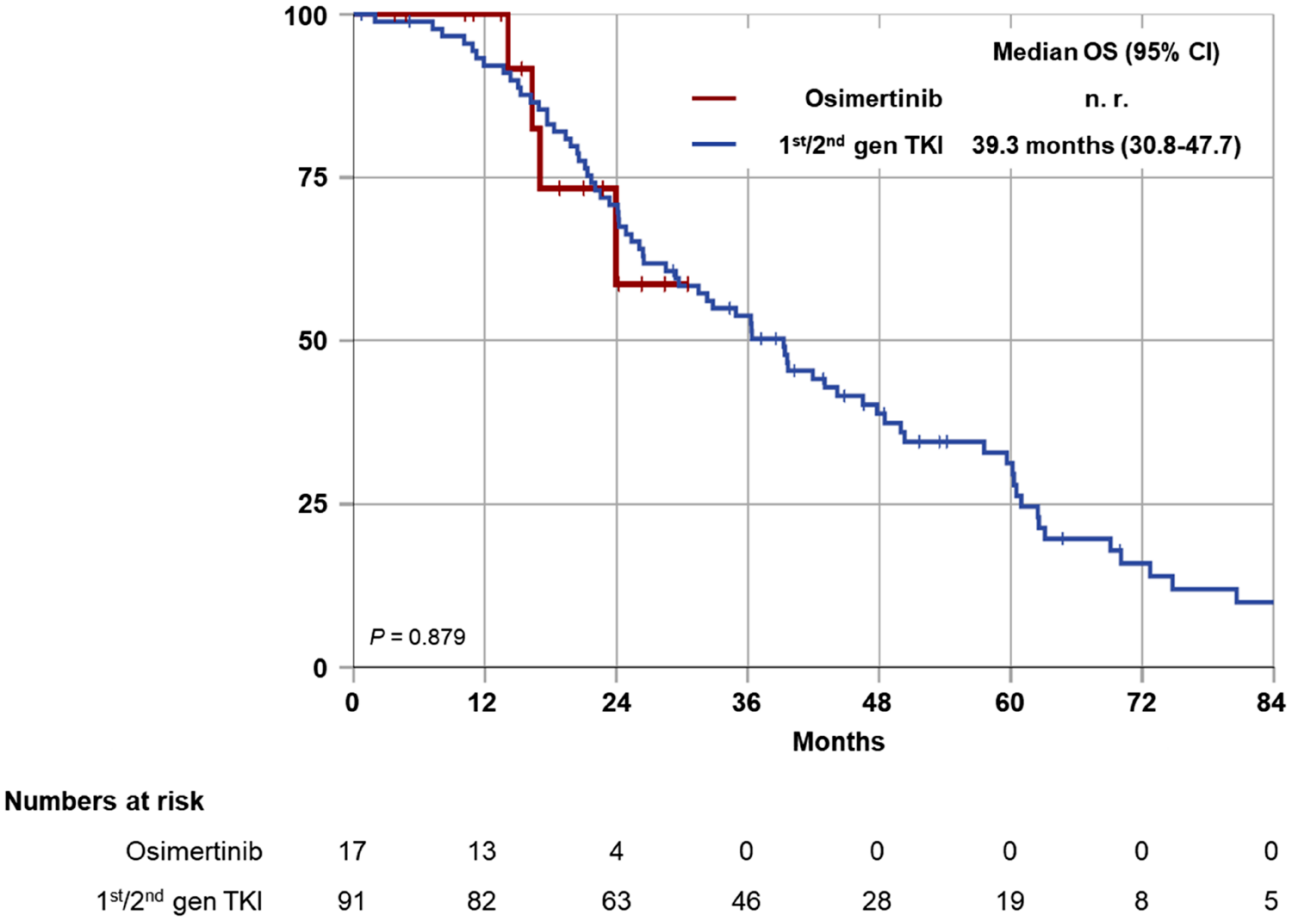
Median overall survival (OS) for patients with first-line 1st/2nd gen TKI vs. first-line osimertinib

Osimertinib was EMA approved for treatment of EGFR p.T790M-positive metastatic NSCLC in February 2016, providing immediate access and reimbursement in Germany. Since February 2016, 91 of the 150 patients were under first-line treatment with a 1st/2nd gen TKI and had therefore the option of second-line osimertinib in case of EGFR p.T790M-positive progression. EMA approval for osimertinib was extended to the first-line setting in June 2018, and 17 of 150 patients received osimertinib as their first TKI. Combined median overall survival (OS) of these two cohorts (*N* = 108) was 39.3 months (95% CI 31.1–47.5 months). There was no survival difference between the cohorts (median not reached vs. 39.3 months, 95% CI 30.8–47.7, *P* = 0.879) (Fig. 3).

### Biomarker analyses at progression and further line treatments

**Fig. 4:**
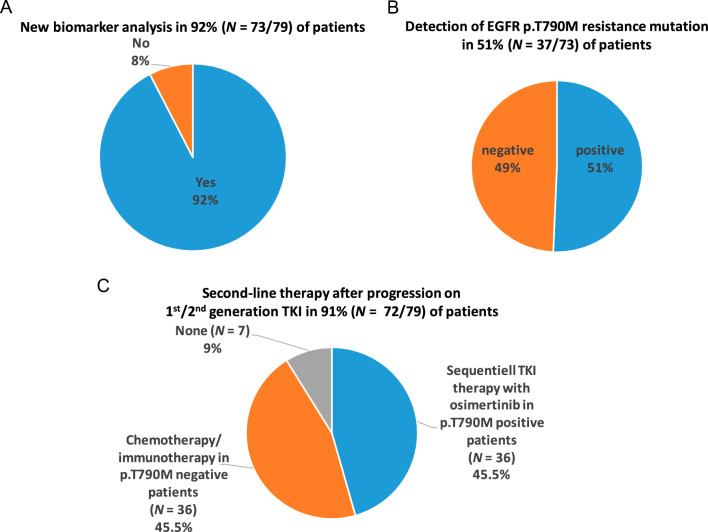
Rate of new biomarker analysis in patients after progression on 1st/2nd gen TKI (**A**). Detection rate of EGFR T790M resistance mutation (**B**). Initiation of second-line therapy in patients progressing on 1st/2nd gen TKI (**C**)

As of March 1, 2021, 79 of 91 of patients had progressed. Of those, 73 patients (92%) underwent a new biomarker analysis (Fig. 4), which identified the EGFR p.T790M resistance mutation in 37 patients (51%).

**Fig. 5:**
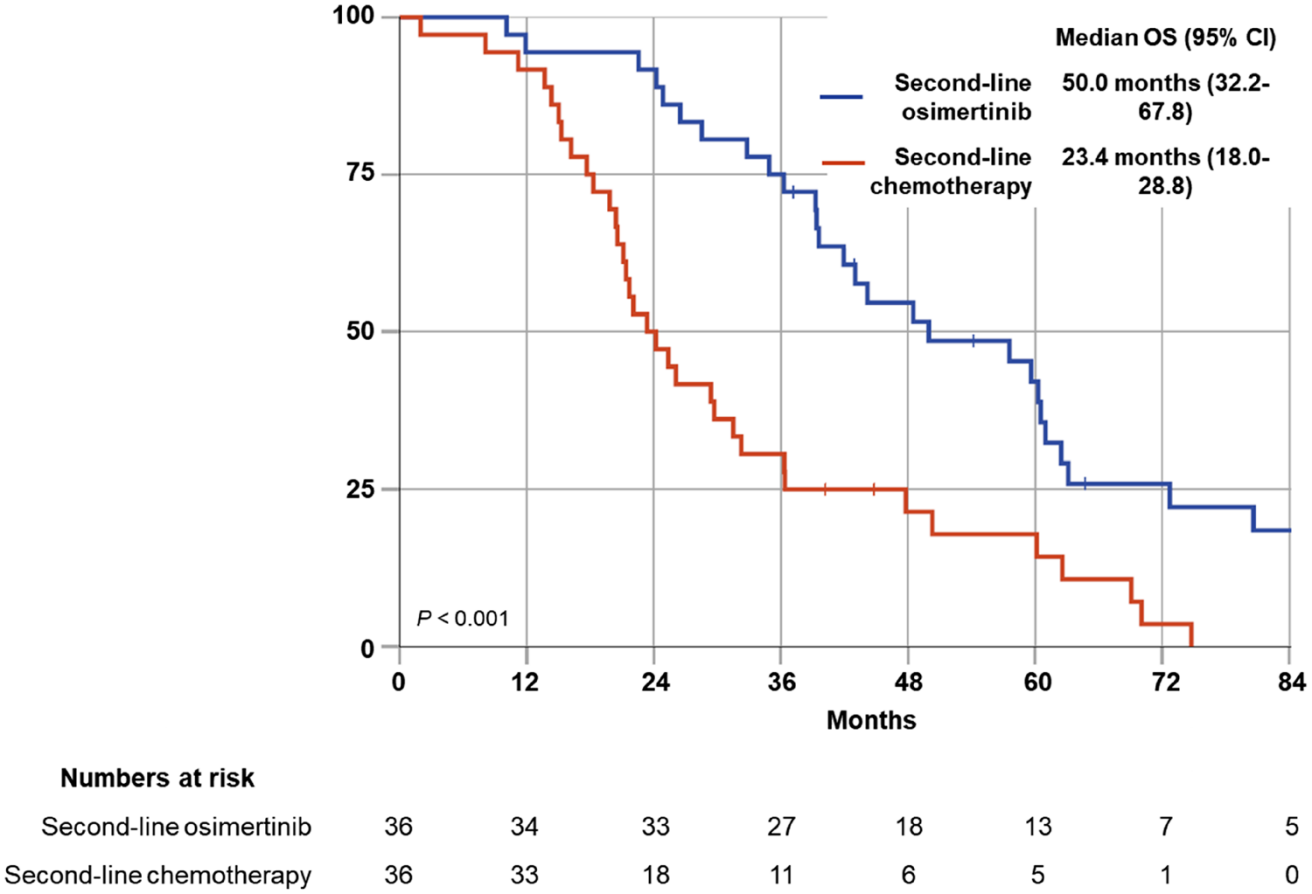
Median overall survival (OS) from the start of 1st/2nd gen TKI for second-line osimertinib in p.T790M-positive patients vs. second-line chemotherapy

In total, 72 of 79 patients (91%) received second-line therapy, including 36 patients with EGFR p.T790M-associated progression. All of those p.T790M-positive patients received second-line osimertinib. Those patients had a superior overall survival as compared to patients receiving second-line chemotherapy for progression with EGFR p.T790M-negative rebiopsy (median 50.0 months, 95% CI 32.2–67.8, vs. 23.4 months, 95% CI 18.0–28.8, *P* < 0.001) (Fig. 5).

Detection of p.T790M resistance mutation in new biomarker analysis after progression on first-line TKI was associated with age < 65 years (*P* = 0.026), common EGFR mutation del exon 19/L858R (*P* = 0.022) and 1st gen TKI (*P* = 0.009) (Table 2).

**Table 2:** Molecular and clinical characteristics for p.T790M-positive vs. p.T790M-negative patients

		p.T790M positive		p.T790M negative		*P* value
		%	*N*	%	*N*	
Age < 65 years		64.9	24	38.9	14	0.026
Age ≥ 65 years		35.1	13	61.1	22	
Gender, female		59.5	22	63.9	23	0.697
Gender, male		40.5	15	36.1	13	
ECOG 0		51.4	19	41.7	15	0.407
ECOG ≥ 1		48.6	18	58.3	21	
Stage at primary diagnosis (IASLC/UICC 8th Edition)						
	I–III	21.6	8	16.7	6	0.591
	IV	78.4	29	83.3	30	
EGFR mutation	Del exon 19/L858R	97.3	36	80.5	29	0.022
	Uncommon	2.7	1	19.4	7	
First TKI						
1st gen TKI		48.6	18	19.4	7	0.009
2nd gen TKI		51.4	19	80.6	29	
Smoking status	Never	37.8	14	41.7	15	
	< = 10 py	24.3	9	22.2	8	
	11–29	13.5	5	16.7	6	
	> = 30	13.5	5	11.1	4	
	n.d	10.8	4	8.3	3	
Curatively intended treatment						
Surgery		21.6	8	19.4	7	
Chemoradiotherapy		10.8	4	13.9	5	
Palliative radiotherapy		73.0	27	72.2	26	

### Treatment after failure of osimertinib

The preferred treatment option after failure of first-line osimertinib was platinum-based chemotherapy in combination with checkpoint inhibitor atezolizumab and anti-VEGF antibody bevacizumab (Table 3). Patients positive for p.T790M and progressing on second-line osimertinib mainly received platinum in combination with pemetrexed. Prognosis after progression on osimertinib was limited with a median PFS for first subsequent therapy after failure of first-line osimertinib of 4.5 months (95% CI 2.0–7.1) and a median PFS of 3.4 months (95% CI 0.9–5.9) for first subsequent therapy after failure of osimertinib in p.T790M-positive patients in the second-line setting. Median OS was 6.9 months (95% CI 4.6–9.2) from the start of first subsequent therapy after progression on first-line osimertinib and 5.6 months (95% CI 3.1–8.2) from the start of first subsequent therapy after failure of second-line osimertinib.

**Table 3:** Treatment after failure of osimertinib in first and second line

Treatment after failure of first-line osimertinib	Pt. no.	Best response	PFS	OS
Atezolizumab + bevacizumab + carboplatin + paclitaxel	1	PR	6.7	10.1
	2	SD	4.5	6.4
	3	SD	3.2	6.9
Cisplatin + pemetrexed	4	SD	5.8	8.7
Median PFS (95% CI)		4.5 months (2.0–7.1)		
Median OS (95% CI)		6.9 months (4.6–9.2)		

Treatment after failure of second-line osimertinib (p.T790M positive)	Pt. no.	Best response	PFS	OS
Cisplatin + pemetrexed	5	PR	6.4	18.1
	6	PR	5.1	5.1
	7	SD	4.7	13.1
	8	SD	1.6	2.2
	9	PD	1.3	1.3
Carboplatin + pemetrexed	10	SD	3.8	6.4
	11	SD	3.4	9.3
	12	PD	2.5	2.5
	13	PD	0.3	0.3
Carboplatin + paclitaxel	14	PD	0.3	0.3
Carboplatin + paclitaxel, followed by pemetrexed maintenance	15	PR	13.6	13.6
Pemetrexed	16	SD	8.3	24.7
	17	PD	0.5	0.5
Afatinib	18	PR	7.7	11.8
	19	SD	5.1	23.5
Nivolumab	20	PD	0.5	5.6
Median PFS (95% CI)		3.4 months (0.9–5.9)		
Median OS (95% CI)		5.6 months (3.1–8.2)		

## Discussion

Osimertinib is currently the most potent TKI for first-line treatment of patient with metastatic NSCLC harboring common (delEx19, L858R) EGFR mutations in terms of progression-free survival (Soria et al. 2018). Currently known mechanisms of acquired resistance to first-line osimertinib are diverse and provide no direct path to a sequenced targeted therapy. Hence, patients progressing under osimertinib are offered chemotherapies, which have limited efficacy in TKI-pretreated patient populations (Mok et al. 2017; Papadimitrakopoulou et al. 2020). These findings are in line with the poor patients’ prognosis in our cohort after failure of osimertinib in the first-line, and in the second-line setting with a median overall survival of 6.9 months (95% CI 4.6–9.2) and 5.6 months (95% CI 3.1–8.2), respectively. Treatment after failure of first-line osimertinib was mainly atezolizumab/bevacizumab/carboplatin/paclitaxel, and platinum/pemetrexed after p.T790M-positive progression on second-line osimertinib. The limited efficacy of chemotherapy-based treatment after failure of osimertinib was underlined by a median PFS of 4.5 months (95% CI 2.0–7.1) and 3.4 months (0.9–5.9), respectively.

The approval of osimertinib is based on the pivotal FLAURA study, which enrolled patients with ECOG 0–1. For patients randomized to the control arm, access to 3rd gen TKIs at progression was high, making OS data relevant. However, the most recent full publication of OS data was still only 58% mature, and a relatively high proportion of patients (30% in both arms) received no post-progression therapy (Ramalingam et al. 2020). Recent studies combining erlotinib with antiangiogenic biologicals leading to approvals have reported PFS rates comparable to osimertinib in FLAURA, while still enabling second-line osimertinib to approximately 50% of patients developing EGFR p.T790M-associated resistance (Nakagawa et al. 2019; Saito et al. 2019). The same is true for studies combining first-line gefitinib with chemotherapy, which led to an improved overall survival compared to gefitinib alone (Noronha et al. 2020). This option is not approved in the EMA legislature. To date, there is no direct comparison of these approaches to first-line osimertinib.

Based on its favorable toxicity profile as compared to TKI targeting wild-type receptors, osimertinib is clearly the first-line TKI of choice for patients less likely to undergo further line therapy, such as frail or comorbid populations. The presence of asymptomatic brain metastases is also used in support of first-line osimertinib. However, treatment decisions are less clear in less comorbid patients with good performance status. Until final OS data and data from studies directly comparing osimertinib with approved 2nd gen TKI (afatinib, dacomitinib), erlotinib/antiangiogenic and gefitinib/chemotherapy combinations become available, selection of the first-line EGFR TKI should be supported by real-world evidence matching the cancer care setting and patient population at the treating center for shared decision making.

Against this background, we have conducted a retrospective analysis of outcomes with TKI therapy in a contemporary cohort of patients treated at two German comprehensive cancer centers. To explore the impact of osimertinib use as second-line TKI in patients with EGFR p.T790M-associated resistance to 1st/2nd gen TKI, we particularly focused on those patients that were under TKI treatment (or initiated TKI treatment) after EMA approval and reimbursement of osimertinib. Overall survival of this population, which received sequenced osimertinib in approximately 50% of patients, was compared and found to be favorable to the overall survival observed in the control arm of FLAURA, which was largely treated following the same algorithm (Ramalingam et al. 2020). Moreover, it also compared favorably to the reported OS of the osimertinib arm of FLAURA. As expected, this good outcome was strongly impacted by those patients acquiring EGFR p.T790M-associated resistance and subsequently receiving osimertinib. These findings are in line with other recently published comprehensive real-world data analysis (Magios et al. 2021). Certainly, selection bias cannot be excluded as patients were treated at two academic comprehensive cancer centers serving densely populated metropolitan areas. On the other hand, our cohort included a high fraction of patients with ECOG PS 2 or 3, who were excluded from FLAURA.

Potentially, careful patient management demonstrated by a rate of 92% of second biomarker analyses in case of progression and a rate of 91% for post-progression therapy might have played a major role in these positive survival outcomes. Nevertheless, it has to be strongly emphasized that patients experiencing EGFR p.T790M-negative resistance unfortunately show a numerically inferior survival as compared to the osimertinib arm of FLAURA (Ramalingam et al. 2020).

Detection of p.T790M in second biomarker analysis after progression on first-line TKI was significantly associated with younger age (< 65 years). Given the relatively small sample size (*N* = 73), these findings should be interpreted with caution. A trend toward a relation between younger age and p.T790M-positive progression was reported in a published retrospective analysis, but did not reach statistical significance (Wu SG et al. 2020). Moreover, detection of p.T790M was positively associated with 1st gen TKI as first-line TKI. Wu SG et al. reported a statistically significant difference in p.T790 detection between 1st gen TKI gefitinib and 2nd gen TKI afatinib, but not between 1st gen erlotinib and 2nd gen afatinib. These findings clearly need validation in larger cohorts and in a prospective fashion. Furthermore, our data show a strong association between common EGFR mutation (del 19, L858R) and development of p.T790M compared to rare EGFR mutations, which is in line with the literature (Yang et al. 2020).

## Conclusion

With our data and the published evidence combined, it appears highly attractive to personalize the first-line TKI selection based on an upfront prediction of the likelihood of developing EGFR p.T790M-mediated resistance. Those patients could benefit by a sequence of a 1st/2nd gen TKI, potentially combined with an anti-VEGF treatment strategy like bevacizumab or ramucirumab, followed by osimertinib. Patients developing other mechanisms of resistance will be better served with first-line osimertinib. Therefore, predictors of p.T790M-associated resistance are needed to optimize first-line treatment decisions. Until such tools have emerged and more definitive data from comparative clinical trials become available, shared decision making on the selection of the first-line EGFR TKI might be based on a comprehensive discussion and risk–benefit evaluation between patients and the treating thoracic oncologist.

## Supplementary Information

Below is the link to the electronic supplementary material.

Supplementary file1 (PPTX 27 KB)

## Data Availability

The datasets generated during and analyzed during the current study are available from the corresponding author on reasonable request.
