# Supplementary material for: Favorable survival outcomes in epidermal growth factor receptor (EGFR)-mutant non-small cell lung cancer sequentially treated with a tyrosine kinase inhibitor and osimertinib in a real-world setting
Source: J Cancer Res Clin Oncol. 2023 May 18;149(11):9243–52. doi: 10.1007/s00432-023-04839-3 (PMC10374675; doi:10.1007/s00432-023-04839-3)
Supplement: Supplementary file 1 — Supplementary file1 (PPTX 27 KB) [file 432_2023_4839_MOESM1_ESM.pptx]

## Slide 1
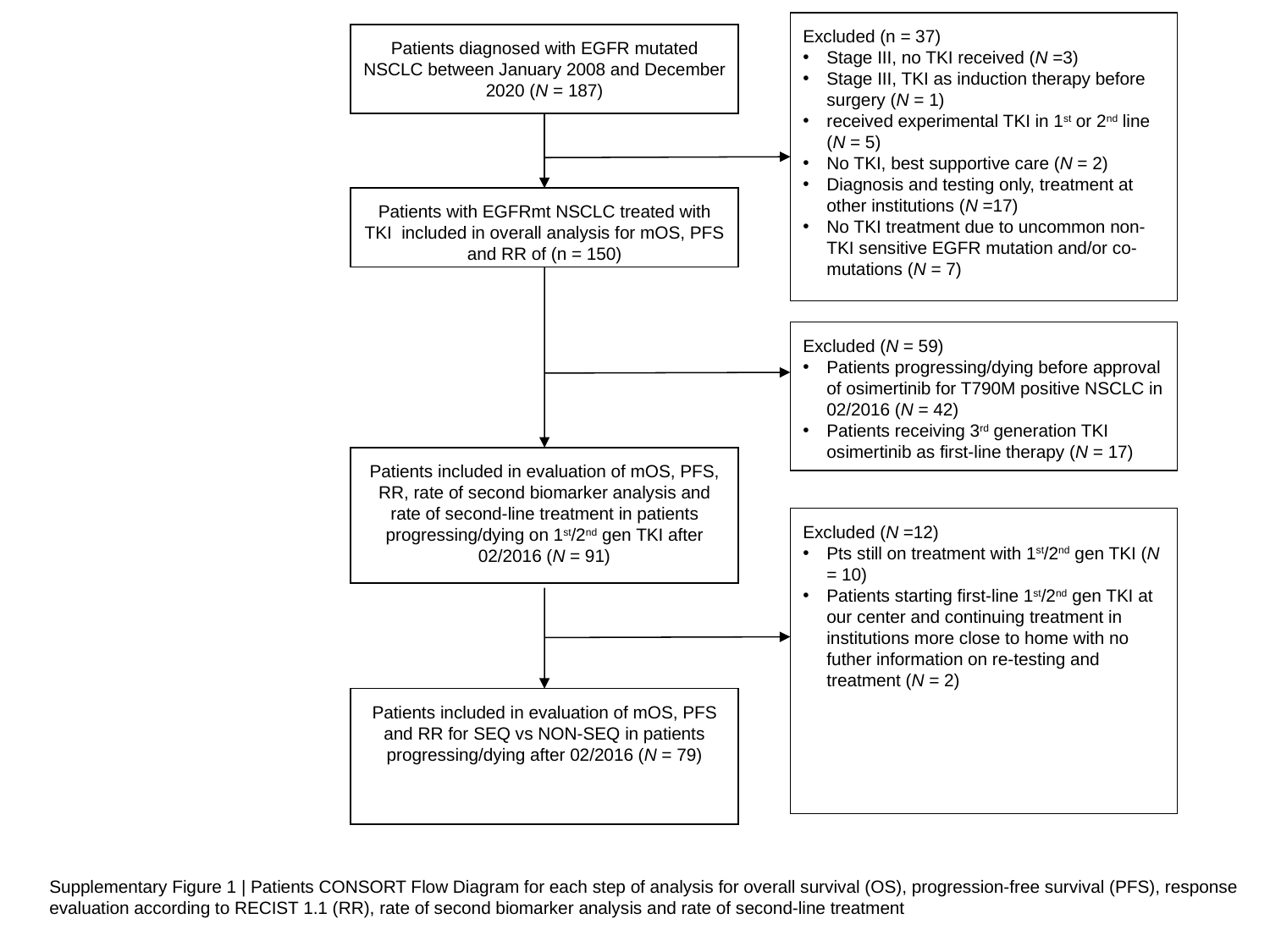

Excluded (n = 37)
Stage III, no TKI received (N =3)
Stage III, TKI as induction therapy before surgery (N = 1)
received experimental TKI in 1st or 2nd line (N = 5)
No TKI, best supportive care (N = 2)
Diagnosis and testing only, treatment at other institutions (N =17)
No TKI treatment due to uncommon non-TKI sensitive EGFR mutation and/or co-mutations (N = 7)
Patients diagnosed with EGFR mutated NSCLC between January 2008 and December 2020 (N = 187)
Patients with EGFRmt NSCLC treated with TKI included in overall analysis for mOS, PFS and RR of (n = 150)
Excluded (N = 59)
Patients progressing/dying before approval of osimertinib for T790M positive NSCLC in 02/2016 (N = 42)
Patients receiving 3rd generation TKI osimertinib as first-line therapy (N = 17)
Patients included in evaluation of mOS, PFS, RR, rate of second biomarker analysis and rate of second-line treatment in patients progressing/dying on 1st/2nd gen TKI after 02/2016 (N = 91)
Excluded (N =12)
Pts still on treatment with 1st/2nd gen TKI (N = 10)
Patients starting first-line 1st/2nd gen TKI at our center and continuing treatment in institutions more close to home with no futher information on re-testing and treatment (N = 2)
Patients included in evaluation of mOS, PFS and RR for SEQ vs NON-SEQ in patients progressing/dying after 02/2016 (N = 79)
Supplementary Figure 1 | Patients CONSORT Flow Diagram for each step of analysis for overall survival (OS), progression-free survival (PFS), response evaluation according to RECIST 1.1 (RR), rate of second biomarker analysis and rate of second-line treatment
